# The Complicated Evolutionary Diversification of the Mpeg-1/Perforin-2 Family in Cnidarians

**DOI:** 10.3389/fimmu.2020.01690

**Published:** 2020-08-06

**Authors:** Brian M. Walters, Michael T. Connelly, Benjamin Young, Nikki Traylor-Knowles

**Affiliations:** ^1^Department of Biology, University of Miami, Coral Gables, FL, United States; ^2^Rosenstiel School of Marine and Atmospheric Science, University of Miami, Coral Gables, FL, United States

**Keywords:** perforin, perforin-2, Mpeg-1, cnidarian, immunity, evolution, phylogenetics, macrophage expressed gene 1

## Abstract

The invertebrate innate immune system is surprisingly complex, yet our knowledge is limited to a few select model systems. One understudied group is the phylum Cnidaria (corals, sea anemones, etc.). Cnidarians are the sister group to Bilateria and by studying their innate immunity repertoire, a better understanding of the ancestral state can be gained. Corals in particular have evolved a highly diverse innate immune system that can uncover evolutionarily basal functions of conserved genes and proteins. One rudimentary function of the innate immune system is defense against harmful bacteria using pore forming proteins. Macrophage expressed gene 1/Perforin-2 protein (Mpeg-1/P2) is a particularly important pore forming molecule as demonstrated by previous studies in humans and mice, and limited studies in non-bilaterians. However, in cnidarians, little is known about Mpeg-1/P2. In this perspective article, we will summarize the current state of knowledge of Mpeg-1/P2 in invertebrates, analyze identified Mpeg-1/P2 homologs in cnidarians, and demonstrate the evolutionary diversity of this gene family using phylogenetic analysis. We will also show that Mpeg-1 is upregulated in one species of stony coral in response to lipopolysaccharides and downregulated in another species of stony coral in response to white band disease. This data presents evidence that Mpeg-1/P2 is conserved in cnidarians and we hypothesize that it plays an important role in cnidarian innate immunity. We propose that future research focus on the function of Mpeg-1/P2 family in cnidarians to identify its primary role in innate immunity and beyond.

## Overview of Cnidaria, Innate Immunity, and Macrophage Expressed Gene 1/Perforin-2 Protein

**Figure 1:**
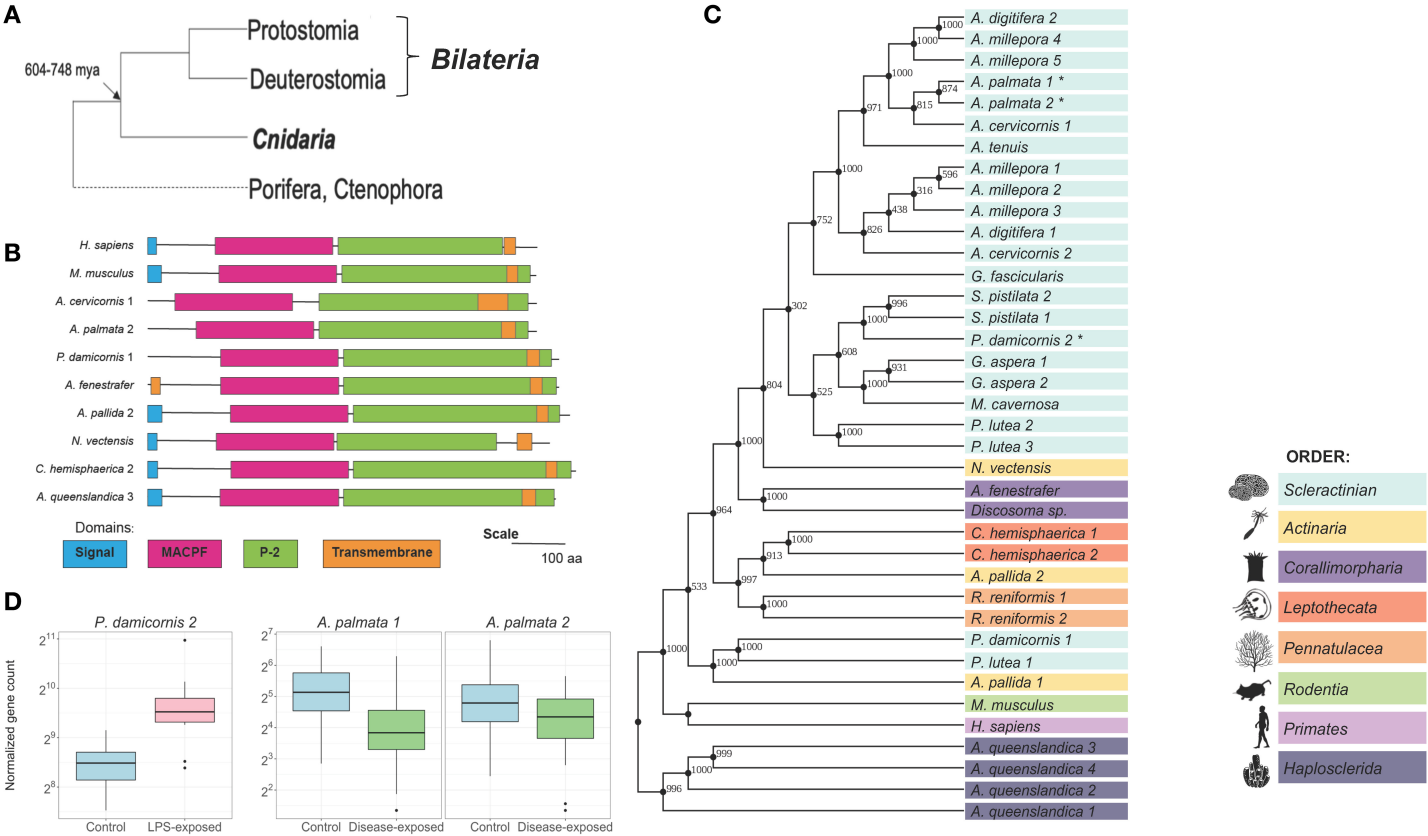
Mpeg-1/P2 is highly diversified in Cnidarians. **(A)** Between 604–748 million years ago, bilaterians split from cnidarians. Cnidaria as sister group to Bilateria can inform our understanding of ancestral traits. **(B)** Domain architecture of P2. Amino acid sequence and domain lengths are drawn to scale. Signal peptide (blue), membrane attack complex/perforin (MACPF; pink), and transmembrane (orange) domains were identified using Hmmer. The P2 (green) domain was identified by aligning the P2 sequence of the human P2 protein. **(C)** A Maximum likelihood tree created in PhyML using the WAG +G +I model, as predicted by Smart Model Selection and ProtTest [Version 3.4.2; (4, 5)]. This tree shows the predicted evolutionary relationship of the whole P2 protein using 38 protein sequences. The percentage of replicate trees associated with the given taxa clustered together using 1000 bootstrap tests are shown at the nodes. All amino acid positions with <95% coverage were eliminated from the model. Tree is rooted taxonomically by *A. queenslandica*. **(D)** The response of Mpeg-1 to an immune stimulus and active white band disease infection in two coral species. In *P. damicornis* LPS exposure causes significant upregulation of a Mpeg-1 transcript (pdam_00017055, Swiss-prot E-value = 7.43E-143) compared with control. In *A. palmata*, exposure to white band disease identified two significantly differentially expressed downregulated Mpeg-1 transcripts (Apalm_v2_evm.model.Sc0a5M3_382_HRSCAF_692.335, Swiss-prot E = 3.55E-158; Apalm_v2_evm.model.Sc0a5M3_382_HRSCAF_692.340 Swiss-prot E-value = 1.69E-159).

The phylum of Cnidaria possesses over 10,000 extant species which are united by the innovation of stinging cnidocyte cells and a polyp life stage (1). Cnidarians include stony corals, soft corals, sea anemones, and jellyfish and are known to be some of the most important organisms for promoting ocean biodiversity, as well as sources of novel compound discovery (2, 3). From an evolutionary perspective, Cnidaria is critical for our understanding of ancestral traits because they are the sister group with Bilateria, from which they split ~604–748 million years ago [Figure 1A; (6, 7)].

The advent of next-generation sequencing technologies has revealed that stony corals possess a highly redundant and diverse innate immune system at the gene and protein levels (8–10). Stony corals maintain symbiotic associations with a diverse microbial community including bacteria, fungi, archaea, and dinoflagellates of the family Symbiodiniaceae (11). To maintain these relationships, they must possess a complex innate immune repertoire that can decipher between symbiont and pathogen (12). These intricate symbioses, paired with old evolutionary age are hypothesized to be the primary factors that have led to the complex diversity of stony coral innate immune proteins (13–15). We currently do not understand the function of many of these innate immune factors, but by examining their phylogenetics, protein domain architecture, and gene expression we can begin to better understand their possible significance.

Macrophage expressed gene 1/Perforin-2 protein (Mpeg-1/P2) is a pore forming effector molecule that is crucial for the innate immune response of both vertebrates and invertebrates (16). It has been identified in multiple organisms, including sponges, mollusks, zebrafish, ctenophores, sea anemones, and humans (16–27). Within invertebrates, limited studies have shown that both sponges and oysters upregulate Mpeg-1 in response to viral or bacterial infections (18, 28). In other invertebrates, including stony corals, little is known about the function or diversity of the Mpeg-1/P2 family. Previous studies have identified MACPF containing proteins in stony corals including within toxins from cnidocyte cells (29–34), however proteins containing both MACPF and P2 domains such as Mpeg-1/P2 have not been well-described.

In this perspective article, we will discuss the diversity of the Mpeg-1/P2 family within Cnidaria, with a focus on stony corals. Additionally, we describe the conserved protein domains of P2 in Cnidaria and show that Mpeg-1 homologs react to both a natural disease challenge, as well as a synthetic pathogen mimic. Lastly, we discuss the possible role of Mpeg-1/P2 in cnidarian innate immunity and future areas of investigation.

## Cnidaria Mpeg-1/P2 Is Highly Conserved, Duplicated, and Complicated

To identify cnidarian homologs, BLASTp (Version 2.2.29+) searches using the *Mus musculus* P2 protein sequence were employed (35). To locate the P2 domain, each sequence was aligned against the isolated *Homo sapiens* P2 domain (amino acids 351–653) using Clustal Omega (36). From this, we identified cnidarian P2 protein homologs as highly conserved (Figure 1B, Supplementary Table 1). This is supported by both protein domain analysis and phylogenetic analysis (Figures 1B,C). There is a diversity of P2 homologs present indicating multiple duplication events within each cnidarian species and thus paralogs. One hypothesis for the retention of multiple P2 paralogs is that it may have evolved additional functions in cnidarians through the process of neofunctionalization. Alternatively, subfunctionalization could have occurred requiring multiple paralogs to perform the original ancestral function.

P2 proteins consist of an N-terminus regulatory signal peptide, the membrane attack complex/perforin (MACPF) domain, the perforin-2 (P2) domain, and the C-terminus transmembrane anchor (16). The MACPF domain generates pores in the lipid bilayer of bacteria cell membranes that leads to bacterial lysis (37). The defining P2 domain is important for Mpeg-1/P2 identification, but little is known about the functional mechanisms of this domain. A single missense or stop mutation in the P2 domain causes an inability to fight off bacterial infections indicating that it is important for the overall primary function of the P2 protein (38).

Using PhyML (Version 3.0) phylogenetic trees were constructed to identify the relationships between cnidarian, mammalian, and sponge P2 proteins (Figure 1C) (39). A maximum-likelihood tree was created using the WAG +G +I model as recommended by both Smart Model Selection and ProtTest [Version 3.4.2; (4, 5)]. The resulting tree shows P2 homologs partitioning into groups based on the major clades of stony corals with Corallimorpharia forming a paraphyletic group sister to the stony corals in accordance with their known evolutionary relationship (40, 41). The 60% of the bootstrap support are over 90% indicating high confidence in our model (Figure 1C).

Taken together these results show that P2 proteins are highly conserved and diverse within Cnidaria, a pattern which has also been observed in other innate immunity genes (13, 14). The phylogenetic relationship of P2 clearly shows that diversification of this protein occurred within species which resulted in many unique paralogs for P2. Given this protein domain analysis and phylogenetic information, understanding if the genes associated with Mpeg-1 are expressed in response to an active infection or a synthetic immune stimulus would further bolster our hypothesis that Cnidaria possesses functional Mpeg-1/P2.

## Stony Coral Mpeg-1 Genes Exhibit Alternate Reactions to Immune Stimulus and Active Infection

Two scleractinian coral transcriptomic datasets were mined for homologs of P2; one from *Pocillopora damicornis* exposed to the synthetic immune stimulus lipopolysaccharide (LPS), NCBI SRA BioProject PRJNA587509, (34) and the second from *Acropora palmata* exposed to the naturally occurring white band disease (WBD); NCBI SRA BioProject PRJNA529682, (42); Figure 1D. In *P. damicornis*, one Mpeg-1 homolog was found to be significantly upregulated (pdam_00017055, LFC = 1.21), while in *A. palmata* two paralogs of Mpeg-1 were found to be significantly downregulated in response to WBD (Apalm_v2_evm.model.Sc0a5M3_382_HRSCAF_692.340, LFC = −1.38; Apalm_v2_evm.model.Sc0a5M3_382_HRSCAF _692.335, LFC = −0.94). The presence of Mpeg-1 significant differential gene expression in these different coral species is evidence that it is responding to bacteria much like what has been previously seen in other organisms, however, unlike these other organisms, the response is more complicated and variable (16–24, 26, 43). With the presence of multiple paralogs, it is possible that some of these genes are not involved in innate immunity. Additionally, the variation in gene expression in *A. palmata* could be due to environmental challenges, as these were nursery reared corals (42). Further investigation into the function of these genes in multiple species of coral will be valuable for our understanding of the functional repertoire of this gene family in cnidarians, as well as, the effects of environmental stress.

## Future Directions for Cnidarian Mpeg-1/P2

The conservation of Mpeg-1/P2, across both the cnidarian lineage and throughout evolutionary history provides evidence that it is an ancient immune factor important for survival. Specifically, within cnidarians, there is much we do not understand: (1) What is the function of Mpeg-1/P2? (2) What is the protein structure? (3) Why are there abundant gene duplications? (4) What other proteins do cnidarian Mpeg-1/P2 associate with? (5) Why do different Mpeg-1/P2 respond differently in distinct cnidarians? (6) In what cell lineage is Mpeg-1/P2 expressed? Investigating Mpeg-1/P2 within non-traditional model systems such as cnidarians will shed light on its full functional capabilities and lead to novel discoveries on the function of this family that could have medically relevant applications.

## Data Availability Statement

Publicly available datasets were analyzed in this study. This data can be found here: https://github.com/brianwalters7/Cnidarian-Mpeg1/tree/v1.3.3.20. *Pocillopora damicornis* sequence data is available through NCBI: SRA BioProject PRJNA587509. *Acropora palmata* sequence data is available through NCBI: SRA BioProject PRJNA529682.

## Author Contributions

BW and NT-K conceived the project and performed the phylogenetic analysis and protein analysis. MC and BY performed the transcriptomic analysis and figure production. All authors were involved in editing and writing of this paper.

## Conflict of Interest

The authors declare that the research was conducted in the absence of any commercial or financial relationships that could be construed as a potential conflict of interest.

## Supplementary Material

Supplementary Table 1 Summary of P2 homologs found in Cnidarians. P2 homologs were identified through BLASTp (Version 2.2.29+) using the Mus musculus protein sequence (NP_001361597.1). Twenty cnidarians genomes, one cnidarian transcript shotgun assembly, and one sponge genome were searched. This included 12 scleractinian corals, one soft coral, two coral-like anemones, three anemones, one hydra, one jellyfish, and one parasitic myxozoa. Candidate homologs from the BLAST searches were limited to E-value cut off (E-6). Sequences were further culled by removing candidates which lacked MACPF or P2 domain. Only three cnidarians (Anemonia viridis, Hydra vulgaris, and Thelohanellus kitauei) did not have homologs present. Lastly, an Orbicella faveolata homolog, was identified, but removed from analysis due to truncation.

## References

[1] Appeltans W Ahyong ST Anderson G Angel MV Artois T Bailly N. The magnitude of global marine species diversity. Curr Biol. (2012) 22:2189–202. 10.1016/j.cub.2012.09.036 23159596

[2] Hughes TP Baird AH Bellwood DR Card M Connolly SR Folke C. Climate change, human impacts, and the resilience of coral reefs. Science. (2003) 301:929–33. 10.1126/science.1085046 12920289

[3] Rocha J Peixe L Gomes N Calado R. Cnidarians as a source of new marine bioactive compounds—an overview of the last decade and future steps for bioprospecting. Mar Drugs. (2011) 9:1860–86. 10.3390/md9101860 22073000 PMC3210609

[4] Lefort V Longueville JE Gascuel O. SMS: Smart Model Selection in PhyML. Mol Biol Evol. (2017) 34:2422–24. 10.1093/molbev/msx149 28472384 PMC5850602

[5] Darriba D Taboada GL Doallo R Posada D. ProtTest 3: fast selection of best-fit models of protein evolution. Bioinformatics. (2011) 27:1164–5. 10.1093/bioinformatics/btr088 21335321 PMC5215816

[6] Ryan JF Burton PM Mazza ME Kwong GK Mullikin JC Finnerty JR. The cnidarian-bilaterian ancestor possessed at least 56 homeoboxes: evidence from the starlet sea anemone, *Nematostella vectensis*. Genome Biol. (2006) 7:R64. 10.1186/gb-2006-7-7-r64 16867185 PMC1779571

[7] Bosch TCG. Cnidarian-microbe interactions and the origin of innate immunity in metazoans. Annu Rev Microbiol. (2013) 67:499–518. 10.1146/annurev-micro-092412-155626 23808329

[8] Palmer CV Traylor-Knowles N. Towards an integrated network of coral immune mechanisms. Proc R Soc B Biol Sci. (2012) 279. 10.1098/rspb.2012.1477 22896649 PMC3441085

[9] Quistad SD Traylor-Knowles N. Precambrian origins of the TNFR superfamily. Cell Death Discov. (2016) 2:16058. 10.1038/cddiscovery.2016.58 27551546 PMC4979521

[10] Traylor-Knowles N Connelly MT What is currently known about the effects of climate change on the coral immune response. Curr Clim Chang Rep. (2017) 3:252–60. 10.1007/s40641-017-0077-7

[11] Rohwer F Seguritan V Azam F Diversity and distribution of coral-associated bacteria. Mar Ecol Prog Ser. (2002) 243:1–10. 10.3354/meps243001

[12] Palmer CV Traylor-Knowles NG Cnidaria: Anthozoans in the hot seat. In: Cooper EL editor. Advances in Comparative Immunology. Cham: Springer (2018). p. 51–93. 10.1007/978-3-319-76768-0_3

[13] Hamada M Shoguchi E Shinzato C Kawashima T Miller DJ Satoh N. The complex NOD-like receptor repertoire of the coral acropora digitifera includes novel domain combinations. Mol Biol Evol. (2013) 30:167–76. 10.1093/molbev/mss213 22936719

[14] Poole AZ Weis VM. TIR-domain-containing protein repertoire of nine anthozoan species reveals coral–specific expansions and uncharacterized proteins. Dev Comp Immunol. (2014) 46:480–8. 10.1016/j.dci.2014.06.002 24933613

[15] Cunning R Bay RA Gillette P Baker AC Traylor-Knowles N. Comparative analysis of the Pocillopora damicornis genome highlights role of immune system in coral evolution. Sci Rep. (2018) 8:16134. 10.1038/s41598-018-34459-8 30382153 PMC6208414

[16] McCormack R Podack ER. Perforin-2/Mpeg1 and other pore-forming proteins throughout evolution. J Leukoc Biol. (2015) 98:761–8. 10.1189/jlb.4MR1114-523RR 26307549 PMC4600061

[17] Mah SA Moy GW Swanson WJ Vacquier VD. A perforin-like protein from a marine mollusk. Biochem Biophys Res Commun. (2004) 316:468–75. 10.1016/j.bbrc.2004.02.073 15020241

[18] Wiens M Korzhev M Krasko A Thakur NL Perović-Ottstadt S Breter HJ. Innate immune defense of the sponge suberites domuncula against bacteria involves a MyD88-dependent signaling pathway: induction of a perforin-like molecule. J Biol Chem. (2005) 280:27949–59. 10.1074/jbc.M504049200 15923643

[19] Miller DJ Hemmrich G Ball EE Hayward DC Khalturin K Funayama N. The innate immune repertoire in Cnidaria - ancestral complexity and stochastic gene loss. Genome Biol. (2007) 8:R59. 10.1186/gb-2007-8-4-r59 17437634 PMC1896004

[20] Wang K-J Ren H-L Xu D-D Cai L Yang M. Identification of the up-regulated expression genes in hemocytes of variously colored abalone (Haliotis diversicolor Reeve, 1846) challenged with bacteria. Dev Comp Immunol. (2008) 32:1326–47. 10.1016/j.dci.2008.04.007 18538840

[21] Wang G-D Zhang K-F Zhang Z-P Zou Z-H Jia X-W Wang S-H. Molecular cloning and responsive expression of macrophage expressed gene from small abalone Haliotis diversicolor supertexta. Fish Shellfish Immunol. (2008) 24:346–59. 10.1016/j.fsi.2007.12.008 18255313

[22] He X Zhang Y Yu Z. An Mpeg (macrophage expressed gene) from the Pacific oyster Crassostrea gigas: Molecular characterization and gene expression. Fish Shellfish Immunol. (2011) 30:870–6. 10.1016/j.fsi.2011.01.009 21272653

[23] Kemp IK Coyne VE. Identification and characterisation of the Mpeg1 homologue in the South African abalone, Haliotis midae. Fish Shellfish Immunol. (2011) 31:754–64. 10.1016/j.fsi.2011.07.010 21803160

[24] Bathige SDNK Umasuthan N Whang I Lim B-S Won SH Lee J. Antibacterial activity and immune responses of a molluscan macrophage expressed gene-1 from disk abalone, Haliotis discus. Fish Shellfish Immunol. (2014) 39:263–72. 10.1016/j.fsi.2014.05.012 24852343

[25] Zakrzewska A van Eikenhorst G Burggraaff JE. Genome-wide analysis of yeast stress survival and tolerance acquisition to analyze the central trade-off between growth rate and cellular robustness. Mol Biol Cell. (2011) 22:4435–46. 10.1091/mbc.e10-08-0721 21965291 PMC3216668

[26] Gorbushin AM. Membrane Attack Complex/Perforin domain-containing proteins in a dual-species transcriptome of caenogastropoda Littorina littorea and its trematode parasite Himasthla elongata. Fish Shellfish Immunol. (2016) 54:254–6. 10.1016/j.fsi.2016.04.015 27094958

[27] Traylor-Knowles N Vandepas LE Browne WE. Still enigmatic: innate immunity in the ctenophore Mnemiopsis leidyi. Integr Comp Biol. (2019) 59:811–8. 10.1093/icb/icz116 31251332

[28] Renault T Faury N Barbosa-Solomieu V Moreau K. Suppression substractive hybridisation (SSH) and real time PCR reveal differential gene expression in the Pacific cupped oyster, Crassostrea gigas, challenged with Ostreid herpesvirus 1. Dev Comp Immunol. (2011) 35:725–35. 10.1016/j.dci.2011.02.004 21371503

[29] Oshiro C Bradley EK Eksterowicz J Evensen E Lamb ML Lanctot JK. Performance of 3D-database molecular docking studies into homology models. J Med Chem. (2004) 47:764–7. 10.1021/jm0300781 14736258

[30] Satoh H Nakamura Y Okabe S. Influences of infaunal burrows on the community structure and activity of ammonia-oxidizing bacteria in intertidal sediments. Appl Environ Microbiol. (2007) 73:1341–8. 10.1128/AEM.02073-06 17189445 PMC1828680

[31] Nagai T Ibata K Park ES Kubota M Mikoshiba K Miyawaki A. A variant of yellow fluorescent protein with fast and efficient maturation for cell-biological applications. Nat Biotechnol. (2002) 20:87–90. 10.1038/nbt0102-87 11753368

[32] Mizuno D Tardin C Schmidt CF Mackintosh FC. Nonequilibrium mechanics of active cytoskeletal networks. Science. (2007) 315:370–73. 10.1126/science.1134404 17234946

[33] Oliveira JS Fuentes-Silva D King GF. Development of a rational nomenclature for naming peptide and protein toxins from sea anemones. Toxicon. (2012) 60:539–50. 10.1016/j.toxicon.2012.05.020 22683676

[34] Connelly MT McRae CJ Liu PJ Traylor-Knowles N. Lipopolysaccharide treatment stimulates Pocillopora coral genotype-specific immune responses but does not alter coral-associated bacteria communities. Dev Comp Immunol. (2020) 109:103717. 10.1016/j.dci.2020.103717 32348787

[35] Altschul SF Gish W Miller W Myers EW Lipman DJ. Basic local alignment search tool. J Mol Biol. (1990) 215:403–10. 10.1016/S0022-2836(05)80360-2 2231712

[36] Madeira F Park Y Mi Lee J Buso N Gur T Madhusoodanan N. The EMBL-EBI search and sequence analysis tools APIs in 2019. Nucleic Acids Res. (2019) 47:W636–41. 10.1093/nar/gkz268 30976793 PMC6602479

[37] Smyth MJ Browne KA Thia KYT Apostolidis VA Kershaw MH Trapani JA. Hypothesis: cytotoxic lymphocyte granule serine proteases activate target cell endonucleases to trigger apoptosis. Clin Exp Pharmacol Physiol. (1994) 21:67–70. 10.1111/j.1440-1681.1994.tb02438.x 8156655

[38] McCormack RM Szymanski EP Hsu AP Perez E Olivier KN Fisher E. MPEG1/perforin-2 mutations in human pulmonary nontuberculous mycobacterial infections. JCI Insight. (2017) 2:e89635. 10.1172/jci.insight.89635 28422754 PMC5396519

[39] Guindon S Dufayard JF Lefort V Anisimova M Hordijk W Gascuel O. New algorithms and methods to estimate maximum-likelihood phylogenies: assessing the performance of PhyML 3.0. Syst Biol. (2010) 59:307–21. 10.1093/sysbio/syq010 20525638

[40] Kitahara MV Cairns SD Stolarski J Blair D Miller DJ. A comprehensive phylogenetic analysis of the scleractinia (cnidaria, anthozoa) based on mitochondrial CO1 sequence data. PLoS ONE. (2010) 5:e11490. 10.1371/journal.pone.0011490 20628613 PMC2900217

[41] Lin MF Chou WH Kitahara MV Chen CLA Miller DJ Forêt S. Corallimorpharians are not “naked corals”: insights into relationships between Scleractinia and Corallimorpharia from phylogenomic analyses. PeerJ. (2016) 4:e2463. 10.7717/peerj.2463 27761308 PMC5068439

[42] Young B Serrano XM Rosales S Miller MW Williams D Traylor-Knowles N Innate immune gene expression in Acropora palmata is consistent despite variance in yearly disease events. bioRxiv. (2020). 10.1101/2020.01.20.912410 PMC7580945 33091033

[43] Benard EL Racz PI Rougeot J Nezhinsky AE Verbeek FJ Spaink HP. Macrophage-expressed perforins Mpeg1 and Mpeg1.2 have an anti-bacterial function in Zebrafish. J Innate Immun. (2015) 7:136–52. 10.1159/000366103 25247677 PMC6738794

